# Collectanea Medica, Consisting of Anecdotes, Facts, Extracts, Illustrations, Queries, Suggestions, &c.

**Published:** 1816-03

**Authors:** 


### COLLECTANEA MEDICA,

CONSISTING OF

ANECDOTES, FACTS, EXTRACTS, ILLUSTRATIONS, QUERIES, SUGGESTIONS, &c.

RELATING TO THE

*History or the Art of Medicine, and the Auxiliary Sciences.*

Quicquid agunt medici,

Nostri farrago libelli.

### MEMOIRS OF THE LATE J. C. LETTSOM, M.D. F.R.S. &c. &c. &c.

*(From Mr. Pettigrew's Eulogy, delivered before the London Philosophical Society, in the presence of his Royal Highness the Duke of Kent, &c. &c. &c.)*

JOHN COAKLEY LETTSOM, one of twins, was born in December 1744, in a small island called little Van Dyke, near Tortola, within the verge of the tropics. His ancestors, on the father's side, originated from Letsom, or, as it is called in Domes-day-book, Ledsom, a small village in Cheshire; on the mother's, side, they are lineally descended from Sir Cæsar Coakley, an Irish Baronet, whose family for many years possessed a seat in the parliament of that kingdom, the last of whom was Sir Vesey Coakley.

At the age of six years he was sent to England for his education, and was placed under the protection of Mr. Fothergill, a celebrated preacher in the Society of Friends, and brother to the late Dr. John Fothergill, who afterwards became his patron. By the former he was entrusted to the care of Mr. (afterwards Dr.) Sutcliff, a name which was never mentioned by Dr. Lettsom but with expressions of gratitude for his friendly attention. After serving the stated time of his apprenticeship, he entered at St. Thomas's Hospital as a dresser. In this situation, the diligence that in after-life so particularly characterized him was productive of several advantages; for many of his fellow students, less careful to obtain the necessary information upon which they are destined to act in their arduous profession, led away by the follies of the day, and more attentive to the seductive allurements of the billiard room, the coffee house, and the theatre, used to entrust their proportion of patients to him, who, by attentive observation, was enabled to enlarge the boundaries of medical science, and to alleviate the miseries of the indigent sufferers.

After two years' study and practice in the hospital, he returned to his native soil, to take possession of the property which came to him by the death of his father, and elder brother, who left very little of the family estate to be inherited by him, except a number of negro slaves, whom, to his eternal honour be it pronounced, he *immediately emancipated.*

At Tortola he settled as a medical practitioner, and by his attention soon obtained an excellent practice, that tempted him to visit the celebrated schools of Paris, Edinburgh, and Leyden, at the last of which universities, on the 20th of June, 1769, he took his degree of M.D. His Thesis was entitled, "Observationes ad Vires Theæ Pertinentes," and was inscribed to Dr. John Fothergill, Mr. Samuel Fothergill, and Dr. Abraham Sutcliff.

After this circuit in pursuit of knowledge, he returned to London, and, under the protection of Dr. Fothergill, commenced practice:—this was in 1769, when he was admitted a licentiate of the Royal College of Physicians, at the head of the list of which his name lately appeared. In 1770, he was elected a fellow of the Society of Antiquaries, and in the following year a fellow of the Royal Society. He also became a fellow of the Linnæan Society.

Many of the charitable institutions in the metropolis originated with himself, and, of those that were planned by others, most received from him considerable improvement, and all his active support. His subsequent marriage with an amiable woman, (still living, and deeply lamenting her irreparable loss) and the addition of a considerable fortune by that marriage, enlarged the means of doing good, nor did the necessary attention to the interests and happiness of a numerous family, (of whom three only are now surviving) the result of that marriage, permit his zeal in the cause of philanthropy to cool, or restrain the current, in very arduous times, of a well-directed liberality. He has, in many instances, fostered genius, cherished science, and expanded the circle of the arts, in periods of individual and national distress, unprecedented in the annals of this country; and his purse, equally with his pen, has been devoted to their cause. Medicine and Botany have been particularly indebted to his zealous researches; foreigners of talents and merit ever found an hospitable reception under his roof; and he constantly kept up a correspondence with the literati of the first eminence, both throughout Europe and America.

Professional men of great practice and popularity, and especially physicians, have more abundant opportunities of becoming useful members of the grand community of mankind, than any other class or order of persons whatsoever. And if, as in the case under our immediate consideration, to great medical practice is added a due proportion of philanthropy, neither the legislator who protects our persons and our property, nor the divine to whom is committed the sacred charge of what is more valuable than the most valued of our worldly possessions, hath so many occasions of administering to the health and the sickness, the strength and weakness of our bodies and our minds.

The talents essentially necessary to this important office, as an excellent author* has remarked, are so extensive, that an accom-

* Wallis.

plished plished physician must have his mind stored with ample materials for the enlargement of his understanding, be led to a diversified cultivation of the polite arts and sciences, conducive to elegance of manners and refined sentiments, and inured to that laborious application, by which distinguished abilities are rendered peculiarly beneficial to mankind.

Dr. Lettsom always considered it to be amongst the foremost of his duties to console the mind, as well as relieve the person of his patient; and, although a press of daily practice made it necessary that he should set a just value on time, he was never governed by the stop watch, to hurry away from the invalid whom he believed might be as much assisted by his society as by his prescriptions. On the contrary, it was his constant practice, (a practice it has often afforded me exquisite delight to witness,) to solace and cheer, by the prevailing aids of gentle and encouraging conversation, as much as by medicine; and he has been known to devote many of those hours necessary to his own repose, to quiet the throbbing pulse, and dispose the wakeful eye of his patient to that sleep, which indeed "ministers to a mind diseased," and so often really "knits up the ravelled sleeve of care."

Among the charitable institutions originated by Dr. Lettsom, or to which he belonged, may be enumerated the General, the Finsbury, and Surry Dispensaries, and the General Sea-Bathing Infirmary near Margate. He was one of the first members of the Philanthropic Society, St. George's Fields, and to the Society for the Discharge and Relief of Persons imprisoned for Small Debts, to the Asylum for the Support and Education of the Indigent Deaf and Dumb, and to the Institution for the Relief and Employment of the Indigent Blind, as well as to several other charitable institutions he was a great contributor. He was one of the celebrated thirty-two who associated with the late Dr. Hawes, and my friend Dr. Cogan, (the author of the elegant Treatise on the Passions,) to support the Royal Humane Society for the Recovery of the Apparently Drowned or Dead.

The Medical Society of London, instituted in 1773, for the promotion of Medical Science, is greatly indebted to our late president. He not only contributed to the increase of its valuable communications, but he also generously presented the society with the house it now occupies. He may, indeed, truly be considered as its father, and liberally he supported it. The library, (which, as a medical one, is, perhaps, for works of reference, the most valuable in the country,) is composed of many rare works presented by him. The society have paid their tribute of respect and gratitude by unanimously adopting the following resolutions:—

"That the Society receive the account of the decease of their "late much valued associate with feelings of deep regret for his "loss—of unfeigned respect for his memory—and of gratitude for "the numerous services rendered by him to the society.

"That the above Resolution be entered in the Minutes, and "subscribed

"scribed by the president, and that a copy be transmitted to his "son, Mr. Samuel Lettsom."

Dr. Lettsom's works of a professional and philanthropic nature are very numerous. On this occasion I shall enumerate only the most important:

1. Reflections on the general Treatment and Cure of Fevers; 8vo. 1772.

2. The Natural History of the Tea Tree, with Observations on the Medical Qualities of Tea, and Effects of Tea Drinking, 4to. 1772.

3. Medical Memoirs of the General Dispensary in London, 8vo. 1774.

4. Improvement of Medicine in London, on the Basis of Public Good, 8vo. 1775.

5. History of the Origin of Medicine, and of the State of Physic prior to the Trojan War. An Oration delivered before the Medical Society, London, 4to. 1778.

6. Hints designed to promote Beneficence, Temperance, and Medical Science, 3 vols. 8vo.

7. An Edition of the Works of J. Fothergill, M.D. in 3 vols. 8vo.

8. A Life of Dr. J. Fothergill, 8vo.

9. The Naturalist's and Traveller's Companion, 8vo. an enlarged edition of which I have, for some time, been engaged in preparing for publication.

His work on the Cow Pock, of which important practice he was an ardent and successful advocate, deserves also to be noticed, and his exposition and conquest of the renowned Mayersbach must also be mentioned. Besides these works, in the Transactions of the Royal Society, of the Medical Society, of the Bath Society, he published many important papers, principally on medical subjects. The chief of his public philanthropic labours were made known to the world through the medium of the Gentleman's Magazine, in which may be found a great number of letters relating to the prisons of the metropolis and various parts of the country.

As a proof of the respect entertained of his various literary labours, it may be observed, that he belonged to no less than sixteen universities. Besides the titles already enumerated, he was a fellow of the Horticultural Society, a fellow of the Medical Society, physician-extraordinary of the City of London Lying-in Hospital, and of the General and Finsbury Dispensaries, a doctor of laws, Cambridge, Massachusetts; honorary member of the Philosophical Societies of Philadelphia, Manchester, and Preston; of the Agricultural Society, Bath; and of the Academy of Sciences, Montpelier. Of the Medical Society he was for several years president. He was a vice-president of a great number of charitable institutions.

The character of a man is, perhaps, best inferred from the general ral tendency of his writings; those of Dr. Lettsom were uniformly philanthropic; every page had for its object, the public good and the elevation of the human character, by the recommendation of the performance of benevolent actions towards the relief of the indigent and sick.

I cannot refrain from introducing to your notice the account of the reclaimed highwayman.

"It was my lot," says the doctor, "a few years ago, to be attacked on the highway by a genteel looking person, well mounted, who demanded my money, at the same time placing a pistol to my breast; I requested him to remove the pistol, which he instantly did; I saw his agitation, from whence I concluded he had not been habituated to this hazardous practice; and I added, that I had both gold and silver about me, which I freely gave him; but that I was sorry to see a young gentleman risk his life in so unbecoming a manner, which would probably soon terminate at the gallows; that, at the best, the casual pittance gained on the highway would afford but a precarious and temporary subsistence, but that if I could serve him by a private assistance more becoming his appearance, he might further command my purse; and at the same time I desired him to accept a card containing my address, and to call upon me, as he might trust to my word for his liberty and life. He accepted my address, but I observed his voice faultered; it was late at night; there was, however, (continues the doctor,) sufficient star-light to enable me to perceive, as I leaned towards him on the window of my carriage, that his bosom was overwhelmed with conflicting passions; at length, bending forward on his horse, and recovering the power of speech, he affectingly said, 'I thank you for your offer,—American affairs have ruined me,—I will, dear sir, wait upon you.'" Two weeks afterwards, a person entered the doctor's house, whom he instantly recognized to be this highwayman: "I come, (said he,) to communicate to you a matter that nearly concerns me, and I trust to your honour to keep it inviolable." The good man told him he recollected him, and requested he would relate his history with candour, as the most effectual means of securing his services; and such was the narrative, as would have excited sympathy in every heart. His fortunes had been spoiled on the American continent, and after a long imprisonment, he escaped to this asylum of liberty, where, his resources failing, and perhaps with pride above the occupation of a sturdy beggar, he rashly ventured upon the most dreadful alternative of the highway, where, in his second attempt, he met with our late president, who finding his narrative literally true, was induced to try various means of obviating his distresses. Dr. Lettsom made application to the commissioners for relieving the American sufferers, but without success; at length, a memorial was presented to the queen, briefly stating his sufferings and the cause of them. Struck with his appearance, pleased with his address, and generously sympathizing with his distresses, her Majesty graciously assured him of patronage, provided his pretensions sions should, on enquiry, be found correct. The result was that in a few days he received a commission in the army, and by his public services, twice did his name appear in the Gazette, among the promotions. After some years of employment in the service of his sovereign, this valuable officer fell a victim to the yellow fever, in the West Indies.

Any observations I could make on conduct like this would be useless—I leave it to your own reflections.

[What follows relates principally to Dr. Lettsom's connection with the London Philosophical Society; after which we have the history of his last illness and death.]

The most painful part of my task now commences—a recital of the circumstances connected with the dissolution of our beloved president. For some time past he had been attending a gentleman professionally—the case proved fatal, and Dr. Lettsom was desirous that the body should be examined; this was chiefly performed by the doctor himself, on the 22d of October. He remained in a cold room for two hours, after which he felt chilly and unwell, but not sufficiently so to excite much alarm. On the 25th I received a note from him, requesting to see me, stating that he had not been ill for twenty-seven years before, that he now had a slight fever, from which he expected to recover in a few days, and that he was fearful it would not be prudent for him to attend the society on the morrow.

On the 26th I visited him, and, alas! found him labouring under a strong rigor—(a severe cold shivering fit) indicative of approaching fever, and complaining of great soreness of his arms, which he considered to be rheumatic. I immediately urged the necessity for great care, and requested he would see his friend, Dr. Babington. He, however, observed that he should be better in a few days, and that he wished for no one to attend him. At that time he had a poor patient resident in White-cross-street, whom he was determined to visit, against which his friends strongly contended, but fruitlessly. He went out (this was on the 27th) and returned literally unable to get out of his carriage, and suffering the most acute pain upon any attempt to be assisted. In the evening he was visited by his friends Dr. Babington and Mr. Norris, and was confined to his room. The next day his disease assumed a more distinct character, and he was unable to move in his bed without assistance, sustaining, with the greatest fortitude, the most excruciating pain. In this situation, his anxiety for his patients was unabated—he requested me to visit them, and was eager to know the progress of their diseases. Perpetual enquiry was directed to this society, and respecting the arrangements for the approaching anniversary, which he was so interested concerning, that he said, provided he was only able to sit, and not even to speak, on that occasion, he would attend it.

On the 30th he appeared improved, but on the 31st great debility came on, attended with slight delirium, which terminated his valuable existence on Wednesday the 1st of November, between three and four o'clock in the morning, without a groan. Thus tranquilly terminated the existence of our much-loved associate and president!

His remains were interred in the Friends' Burial Ground, Little Coleman-street, Bunhill-row, on Tuesday the 7th of November.

---

Having in a former Number, under this division of our labours, given some account of a case of Elephantiasis in St. Bartholomew's Hospital, we shall transcribe a description of the same from two documents; they have appeared before the public. The first is from the Transactions of the Medico-Chirurgical Society, in which also another case is inserted; the second from the Medical Transactions of the Royal College of Physicians of London.

"*Two Cases of the True Elephantiasis, or Lepra Arabum.* By William Lawrence, Esq. F.R.S. Professor of Anatomy and Surgery to the Royal College of Surgeons, &c. &c.; and H. H. Southey, M. D. Physician to the Middlesex Hospital.

"The tubercular elephantiasis has been so seldom seen in England, that no case of it had occurred under the observation of my friend Dr. Bateman, when he published his practical Synopsis, a work highly honourable to the medical literature of this country, and constituting an important æra in the cutaneous department of nosology. Having stated to the society, in conversation, some particulars concerning a boy in St. Bartholomew's Hospital, whose case presented the uncommon circumstances of elephantiasis occurring in an individual of English parentage, almost beginning in England, and going through its various stages to an apparent cure in this country; and concerning a female patient under the care of Dr. Southey, whom I had twice seen, I was requested by the president to procure for the society a written account of the disease, as it appeared in these cases. The following narratives have been drawn up by Dr. Southey and myself, in compliance with this request.

"Case I.—Charles Uncle, aged 14, of a dark complexion, with brown hair and dark iris, was admitted into St. Bartholomew's Hospital on the 1st of April, 1814. From himself and his grandmother I learned the following circumstances of his history. His father and the whole of his father's family were English: his mother was born in America, of English parents. They married in England very young, went to settle in America, and had three children born at Augusta, in the United States. The eldest boy and a girl were sent to England young; neither of them ever had any cutaneous complaint. The boy is now alive and in good health; the girl died at the age of sixteen, of consumption after measles. Charles, the youngest child, having lost his father, was removed in early infancy to New Providence in the Bahama Islands, where he lived till the year 1813. He was obliged by his father-in-law to work hard in the open air, exposed to the weather and particularly to the heat of the sun, receiving a sufficient quantity of food, which he described as being of a coarse kind, the same that was given to the negroes. Among the latter, or among the white inhabitants of New Providence, (which is a sea-port,) he never remembers having seen any complaint like his own. In the autumn of 1813 he left New Providence, in perfect health, for England. Being obliged to work in the ship during his passage, he became on one occasion extremely wet, and took a violent cold. He felt himself ill and very drowsy, but his appetite did not fail. In a short time his head and face swelled prodigiously: this swelling gradually subsided, he felt himself better, and tubercles of the skin began to appear in the ears and face; a stiffness of the limbs came on at the same time, and has continued ever since. He arrived in England in the autumn of 1813. The disease, which began in the head, had appeared in various parts of the upper and lower limbs by the month of April, 1814, when he was received into St. Bartholomew's Hospital; but the trunk has always been completely free.

"The disease has every where begun with flattened tubercular elevations of the skin, not larger than half a small pea at first, but increasing afterwards, in some parts to a much more considerable size. Their colour and consistence hardly differed, at their first production, from those of the sound skin; but they soon became red, and acquired in some instances a deep tint of that colour, with a rather livid cast. In some parts they remained in this state: in others an abundance of white and small scales was formed. Some of the tubercles cracked and ulcerated; but the ulcerations were not in general deep or extensive: they furnished a matter which concreted into hard crusts, and caused the dressings to stick very firmly.

"The progress of the complaint was not attended with pain, except from these fissures and ulcerations.

"At the time of his admission into the hospital, this boy's ears, forehead, eyebrows and eyelids, and indeed the whole face, were completely occupied by the disease. The ears and other features were greatly deformed: the former exhibited some of the largest and reddest tubercles, and had suffered the greatest alteration of figure. The nose was flattened and expanded laterally; the lips and cheeks swollen; the hair of the eyebrows dropped off; but although the eyelids were tuberculated, even on their edges, the cilia have not been lost. The hairy scalp was never affected, nor has its hair fallen off. The membrane of the palate and the velum palati were tuberculated, but never, I believe, ulcerated; nor was swallowing impeded. There was no reason to suppose that the bones or membranous lining of the nose participated in the disease. The voice was rather rough and hoarse. The fingers, hands, and wrists, particularly the backs of them, were occupied by numerous tubercles, which reached for a short distance on the fore-arms. A small crop occupied the anterior convexity of the shoulder on each side. The toes and feet were swelled altogether; the under surface red and tubercular. The back of the foot and ankle were affected, and a few scattered tubercles appeared on the thighs.

"Whether there was any unnatural fulness in the upper and anterior part of the thigh might be doubted; but certainly there was no decided swelling in the situation of the 'femoral tumour,' described by Dr. Adams, in the cases of elephantiasis, observed by him on the island of Madeira. The boy was not aware that any change had occurred in this part, or that it was at all enlarged: and at present there is not the slightest appearance of any swelling. An inguinal gland on each side was rather more distinct than usual.

"The condition of the generative organs corresponded with the description of Dr. Adams just alluded to. Not only had their developement been arrested from the time when the disease broke out, but they had actually undergone diminution and decay. The scrotum was shrivelled and seemed empty; the testes could with difficulty be felt; they were soft and about the size of small horse-beans.

"His general health was hardly affected, the appetite being good, the tongue clean, the functions of the bowels regularly performed; and he slept well.

"During the first part of his residence in the hospital the disease advanced. New tubercles appeared on the ears, face, and hands; the two former parts became greatly swollen, and occupied by painful ulcerations. The incrustations of the discharge occasioned difficulty and pain in the motions of speaking and eating. The ulcerations in the face were never deep: they healed in one part and broke out in another. A few deeper ulcers formed at one time on the wrist; they appeared as if a piece of the skin had been dug out, leaving a smooth red surface.

"Of local applications, mild ointments and emollient poultices were the most beneficial, particularly when there was any irritation or inflammation. By loosening the crusts, and softening the parts, they gave ease.

"Various internal means were employed, as mercury, antimony, and arsenic. They disturbed his health, which seemed to aggravate the complaint: this was particularly the case with arsenic. When he left it off, and took sulphuric and nitric acids, he was evidently relieved. Medicines were so obviously inefficacious, to say the least, that attempts at cure by means of the materia medica were not continued; and there is no ground for ascribing the modification which the complaint afterwards underwent to its agency. He took acids and tonics for some time, and such occasional remedies as circumstances required. He was allowed a full diet of meat, porter, and wine.

"He had a very well marked attack of shingles, (herpes zoster,) accompanied by the usual feverish symptoms, which confined him to bed for a few days. The vesicles extended from the linea alba to the spine, on one side of the abdomen, and were numerous and confluent.

confluent. He also went through the measles, which he contracted from a patient in the same ward: the disorder was mild.

"The elephantiasis having been for some time stationary, began to decline about the end of December: the ulcerations healed, all the tubercles lessened, and at last disappeared, and the patient was discharged from the hospital on the 2d of February, 1815. There was at this time no trace of tubercles in the face; but it presented cicatrices, the remains of the ulcerations. The skin had become smooth and soft, excepting so far as its surface was irregular from the scars, and had recovered its natural colour. The features are of course permanently deformed, the lips in particular contracted and turned in, so as to narrow the opening of the mouth; and the cuticle continues to separate from them in dry flakes. Vestiges of the tubercles are visible on the palate and throat, but the uvula is entire. The ears are still thickened and swoln, though much reduced from their former size. The tubercles have disappeared from the extremities, leaving however some cicatrices and roughness of the skin. The toes and soles of the feet are still unnaturally red and swoln, and the legs are altogether tumid, and œdematous towards the lower parts; some indurations are felt in them under the skin.

"While this amendment has proceeded on the outside, there is reason to fear that some internal organs have become affected. As the tubercles have disappeared, a cough has arisen, which is now troublesome.

"The boy is short-breathed and weak, and his pulse is from 110 to 120. He is also much emaciated. The generative organs continue in the state described.

"Charles Uncle went from St. Bartholomew's Hospital to Brompton, where I saw him, after a short interval, labouring under the symptoms of pulmonary affection already mentioned to a considerable degree, and indulging freely in the use of porter, meat, &c. with the view of restoring his strength. I recommended a change of diet, and that he should immediately go into Devonshire, where some of his relations resided. The following is part of a letter from him, dated the 9th of May. 'My bodily health is much improved with respect to strength and eye sight; but I have within a week broken out in three or four places about my face, which I think is merely change of climate. It does not bear the same appearance with the old complaint, as it looks raw when the scabs fall off. I am, according to your advice, placed at a farm-house, where I am comfortable. I amuse myself with shooting, and fishing, and reading.' By a letter from his mother, of the 22d of June, I understand that his face continued very bad at that time, and I was informed at the end of August that it was still broken out, although his health and strength were considerably mended. His brother died of consumption about this time."

"*Case II.* By Dr. Southey.

"In January 1814, I was first requested, by my friend Mr.

Ashburner, to visit a patient of his afflicted with elephantiasis. Of the nature of the complaint no doubt could be entertained, as the symptoms were too strongly marked to be mistaken; and many eminent practitioners both in England and India had agreed in the name, though they had not succeeded in removing the disease.

"Miss R—— is at present twenty-two years of age, she is a native of Bombay, the daughter of an English officer, by a Hindoo woman. When about ten years old, red blotches appeared upon different parts of her body, which by mercurial medicines and other remedies were removed for a time, but recurred at intervals during several years. The elbow and the foot were the parts first attacked by the tubercular disease, which has now existed above five years. These tubercles vary in size, are of a livid colour, and the skin is thickened in their vicinity (indeed upon the feet and hands this thickening of the skin seems to precede their formation): they enlarge gradually with little or no pain, and suppurate. The ulcers thus formed spread along the integuments, laying bare the muscles. The edges of the ulcers are in general high, callous, and jagged. The hands, arms, and legs, are at present nearly covered with ulcerations of this description. The face is also horribly disfigured: the eye-brows have their former situation marked by lines of scurf. The eye-lids are livid and tuberculated, but some few eye-lashes still remain. The alæ nasi are thickened, and the nose quite flat. Dry black crusts, open ulcers, or tubercles advancing to suppuration, cover nearly the whole face. The ears have that thickening of the lobe peculiar to this disease, and are otherwise enlarged and altered in shape. The lips and tongue are studded with small tubercles, and the tonsils commonly more or less ulcerated. A part of the uvula has disappeared. The voice is hoarse. Small tubercles have also formed on the tunica conjunctiva, one so near the edge of the transparent cornea as to have occasioned an opacity of that membrane, and the left eye is beginning to suffer in a similar manner. The trunk is not affected. All the ulcers have at different times been healed, but fresh tubercles constantly form, and go through the same process. According to the state of the general health, the existing ulcers either spread or put forth healthy granulations. The pulse is always quick, varying from 100 to 120. The appetite is weak and the digestive organs torpid, constant purgatives being required. The mentrual discharge is said to have been tolerably regular in point of time; but it was found by Mr. Ashburner to coagulate upon exposure to air. With regard to the libido inexplebilis, or the absence of sexual passion; it may be proper to state, that an offer of marriage was made to this unfortunate female within the last two years, which she was inclined to accept. I have not ascertained the presence of the femoral tumour, but I understand that Dr. Adams upon examination found this mark of the disease on the left thigh. The breasts have disappeared. Among the many unsuccessful remedies which have been tried in this case, acids and alkalies, vegetable and mineral tonics, arsenic, dulcamara and sarsaparilla may be be mentioned. From some combinations of antimony and mercury, particularly that of pulvis antimonialis with the blue pill, temporary advantage seems to have been derived. Of the local remedies, the alternation of poultices with bandages of adhesive plaster was found by Mr. Ashburner to answer best."

The following is Dr. Roberts's account of the case given above by Mr. Lawrence.

"Charles Uncle, between 14 and 15 years of age, came under my care in St. Bartholomew's Hospital, June 7, 1814, and, from his relation, it appeared that he was born at Augusta, in Georgia, North America, of English parents, who were healthy, and his ancestors hardy and robust; that, soon after his birth, he was taken to the Bahama Islands, was latterly employed on-board a ship, and about a year ago was sent to England as a cabin-boy; that during the voyage, after being obliged to sleep on the deck, exposed to rain for three successive nights, he felt heavy and unwell, and soon afterwards his head began to swell, his eye-lids and the lobes of his ears to enlarge, and tubercles to appear over the face; his breath became offensive, his urine thick, and his genitals diminished in size, from which also the hair disappeared, as well as from the eye-brows and arm-pits; that, after his landing, some tubercles were formed also on the hands and feet, and he confessed, what might reasonably be expected, that he had no sexual desires.

"His countenance was much deformed by these elevations; his lips were swollen, and became intersected with fossæ; the alæ of the nose were enlarged, flattened, and scabrous, so as somewhat to impede respiration, and from the disease extending to the throat, particularly over the uvula and tonsils, the gums being at the same time tumid and highly vascular, the voice was weak and hoarse; there were, besides, glandular tumours near the groin on both sides.

"The tuberculated parts were nearly of the same colour as the rest of the skin, and were so insensible, that scarification, which had been practised upon some of them, had hardly given him any pain; and the greatest inconvenience he felt was from the itching of the affected parts, from some of which a sanious fluid was discharged, succeeded by a scab: on others a superficial ulceration took place, and from many a furfuraceous scaling of the cuticle. He complained at times of head-ach, and observed that he never perspired; but in other respects said that he was not unwell, having a very good appetite and regular bowels. It appeared that he had never seen any one affected with a similar disease.

"This was clearly a confirmed case of elephantiasis Græcorum (lepra Arabum) so accurately described by Dr. T. Heberden, in the first volume of the Medical Transactions, as it occurs in the Island of Madeira, and lately further illustrated by Dr. Adams.

"It appeared that this patient had already taken liquor arsenicalis without success, and I directed acid nitric. ex decoct. sarsap. comp.

comp. in large and repeated doses, with the full diet, and a pint of porter daily; and under this plan of treatment, the disease sensibly diminished, but, soon afterwards increasing, he took hydrarg. submur. gr. ½ cum extract. conii gr. v. every night, in addition to the former prescription, but without any good effect. Trial was then made of acid. phosphor. and afterwards cinchona cum potass. nitrat. decoct. dulcamaræ & pil. hydrarg. submuriat.; also acid. sulphuric. with no abatement, but rather an increase, of the disorder.

"Seeing, therefore, little probability of relieving my patient by medical treatment alone, and that his powers rather declined, his appetite continuing at the same time unimpaired; and reflecting that the lower class of persons were chiefly the subjects of this complaint, occurring, as it frequently does, in catholic states, where meagre days are observed, I directed for him, October 4, a full and invigorating regimen, consisting of a pound of meat, with the usual allowance of bread, &c. a pint of Port wine, and a pint of porter daily, which he continued for some months, when, after taking decoct. cinchon. cum tinct. cinchon. comp. every six hours, on account of a herpetic eruption which appeared on the abdomen, attended with fever, and which was soon relieved, he appeared with a most striking general improvement in the tubercular disease; and, though afterwards affected with a diarrhœa and some cough, he still continued to make progress towards recovery from the original complaint, and with the assistance of hydrarg. submuriat. gr. j. cum rhei gr. v. et opii gr. ¼ every other night, and the vapour of hot water to the face, and warm fomentations to the extremities, the whole of the tubercles, becoming softer, subsided, and the surface at length healing, he exhibited an entire new countenance, so that I scarcely knew him; and on February 10, 1815, was well enough to be discharged from the Hospital, in order that he might go to his parents in Devonshire.

"I have been able to find but one case of this disease, which is recorded in the paper of Dr. T. Heberden before alluded to, where a similar result of the softening and healing of the tubercles took place, which both physician and patient attributed to an electuary composed of three parts of cinchona and one of sassafras, taken in the quantity of a large nutmeg, night and morning; a regular, and probably more nourishing diet, than the patient had been accustomed to, being at the same time allowed.

"This complaint seems to be no where generated but in tropical climates; and from the insensibility of the tuberculated parts, does not appear to extend, in its primitive state, below the rete mucosum; but such important deviations from the natural state can hardly exist without some general cause of disease, so difficult of removal, indeed, as usually to afford a case, where it most frequently occurs, hopeless as to its event, and almost disregarded."

CRITICAL

